# Antarctic Krill Are Reservoirs for Distinct Southern Ocean Microbial Communities

**DOI:** 10.3389/fmicb.2018.03226

**Published:** 2019-01-15

**Authors:** Laurence J. Clarke, Léonie Suter, Robert King, Andrew Bissett, Bruce E. Deagle

**Affiliations:** ^1^Antarctic Climate and Ecosystems Cooperative Research Centre, University of Tasmania, Hobart, TAS, Australia; ^2^Australian Antarctic Division, Kingston, TAS, Australia; ^3^Commonwealth Scientific and Industrial Research Organisation, Hobart, TAS, Australia

**Keywords:** Antarctic krill, *Euphausia superba*, Southern Ocean, microbiome, high-throughput DNA sequencing, 16S rRNA

## Abstract

Host-associated bacterial communities have received limited attention in polar habitats, but are likely to represent distinct nutrient-rich niches compared to the surrounding environment. Antarctic krill (*Euphausia superba*) are a super-abundant species with a circumpolar distribution, and the krill microbiome may make a substantial contribution to marine bacterial diversity in the Southern Ocean. We used high-throughput sequencing of the bacterial 16S ribosomal RNA gene to characterize bacterial diversity in seawater and krill tissue samples from four locations south of the Kerguelen Plateau, one of the most productive regions in the Indian Sector of the Southern Ocean. Krill-associated bacterial communities were distinct from those of the surrounding seawater, with different communities inhabiting the moults, digestive tract and faecal pellets, including several phyla not detected in the surrounding seawater. Digestive tissues from many individuals contained a potential gut symbiont (order: Mycoplasmoidales) shown to improve survival on a low quality diet in other crustaceans. Antarctic krill swarms thus influence Southern Ocean microbial communities not only through top-down grazing of eukaryotic cells and release of nutrients into the water column, but also by transporting distinct microbial assemblages horizontally via migration and vertically via sinking faecal pellets and moulted exuviae. Changes to Antarctic krill demographics or distribution through fishing pressure or climate-induced range shifts will also influence the composition and dispersal of Southern Ocean microbial communities.

## Introduction

Marine microbial assemblages play key roles in global biogeochemical cycling (Sunagawa et al., 2015). However, the contribution of macro-organisms to marine microbial assemblages is often overlooked (Tang et al., 2010; Troussellier et al., 2017). Macro-organisms represent nutrient-rich and potentially low-oxygen environments compared to the surrounding water column, as well as providing both internal and external surfaces for microbial colonization. The diversity of niches provided by macro-organisms helps maintain marine microbial diversity and disseminate rare microbes (Troussellier et al., 2017).

Antarctic krill (*Euphausia superba*) is a keystone species of Southern Ocean food webs (Croxall et al., 1999), with an estimated biomass of 379 million tons (Atkinson et al., 2009). Antarctic krill represent nutrient-rich microenvironments in the Southern Ocean, supporting bacterial abundances several orders of magnitude higher than the surrounding seawater (Donachie and Zdanowski, 1998). Antarctic krill, like other zooplankton, are likely to support distinct bacterial communities compared to seawater (Tang et al., 2010; Troussellier et al., 2017). Given their high abundance and circumpolar distribution, krill-associated microbiota likely make a substantial contribution to Southern Ocean microbial communities.

Antarctic krill-associated microbiota are also likely to vary between tissue types due to the different environments they represent. Krill moults are likely to be colonized by bacteria with chitinase activity such as *Colwellia*, *Alteromonas* and *Vibrio* sp. (Cottrell et al., 2000; LeCleir et al., 2004). Zooplankton gastro-intestinal tissues and faecal pellets represent oxygen-depleted environments able to support obligately anaerobic bacteria (Marty, 1993; Proctor, 1997). Furthermore, tissue-specific bacterial communities may be influenced by other factors such as geographic location and developmental stage. Southern Ocean bacterial communities are known to vary between water masses (Wilkins et al., 2013), which will influence the bacteria available to colonize internal and external surfaces. Changes in diet with either location, season (and food availability) or developmental stage (e.g., Schmidt et al., 2014) will alter both the bacteria ingested and the nutrient environment within the zooplankton gut.

We used high-throughput sequencing of the bacterial 16S rRNA gene to characterize seawater and krill-associated bacteria from four locations in the Southern Indian Ocean. We hypothesized that krill-associated microbiota would be distinct from the surrounding seawater, and that different krill microhabitats (moult, stomach, digestive gland and faeces) would harbor distinct communities. We also tested whether geographic location, developmental stage, or sex could explain the observed variation in bacterial communities for each microhabitat.

## Materials and Methods

### Sample Collection

**FIGURE 1:**
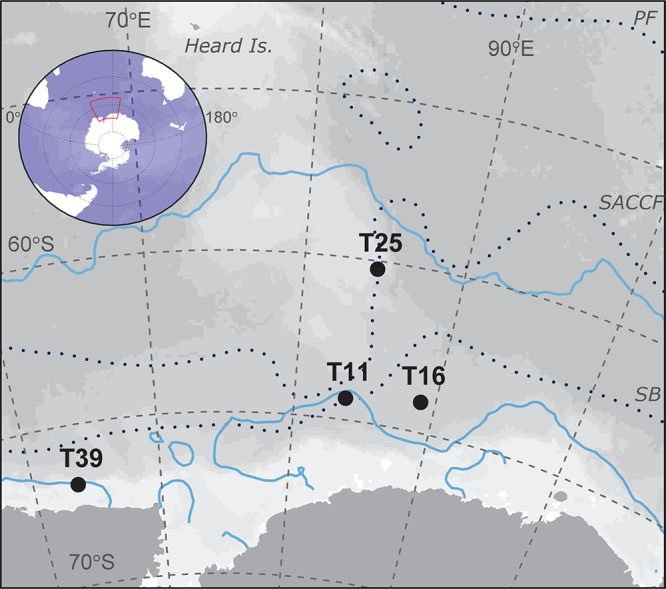
Trawl locations used in this study of krill-associated bacteria. The distance between the closest trawls (T11 and T16) is 260 km and the furthest trawls (T25 and T39) is 1269 km. Upper and lower blue lines show the October and January sea ice extent, respectively. The mean locations of the principal fronts (following Orsi et al., 1995) are shown as dotted lines. PF, polar Front; SACCF, southern Antarctic circumpolar current front; SB, Southern boundary of the ACC.

Samples were collected on board the *RSV Aurora Australis* during cruise V3 between 31 January and 19 February 2016 as part of the Kerguelen Axis voyage. The physical oceanographic context for the voyage is described in Bestley et al. (2018). Antarctic krill were sampled from four swarms in the Indian sector of the Southern Ocean (Figure 1) using targeted trawls with a Rectangular Mid-water Trawl 1+8 (RMT-1+8 m^2^) net. Trawl depths ranged between 10–25 and 65–80 m (determined by acoustically estimated depth of the swarm in the water column), and the pairwise distance between trawls ranged from 260 to 1269 km. Immediately after each trawl, two liters of surface water (4 ± 2 m depth) from the vessel’s uncontaminated seawater line was filtered onto 0.22 μm Sterivex filters and stored at −80°C to compare seawater and krill bacterial community profiles.

In order to isolate moult, faecal and tissue microbiomes, live krill, immediately after capture, were transferred to 250 mL jars (one krill per jar) which were ventilated with small holes to allow seawater exchange as per Virtue et al. (2010). The jars were incubated in large (1600 L) flow-through seawater tanks close to ambient ocean temperature (approx. 1°C) with no additional food provided (Kawaguchi et al., 2006). Jars were inspected for moults at 12 h intervals, with the first six animals to moult from each trawl sampled for microbial community profiles (all collected within 48 h). The animal, associated moult, and any faecal material present were removed from the jar, rinsed with 0.22 μm-filtered seawater and stored separately in liquid nitrogen before being stored at −86°C on return to Australia. For one trawl (T16), seawater from the six individual jars was filtered separately onto 0.22 μm Sterivex filters to test whether moult and faecal bacterial community profiles differed from their experimental environment.

On return to Australia, sampled krill were thawed and the stomach and digestive gland dissected, and rinsed with 0.22 μm-filtered seawater. Animals were staged/sexed and the length of the uropod exopodites recorded as a proxy for animal size as per Virtue et al. (2010) either on the ship or immediately prior to dissection (Melvin et al., 2018).

### DNA Extraction, PCR Amplification and High-Throughput Sequencing

Tissue samples (moult, stomach, digestive gland, faeces), Sterivex filters and two extraction controls (1 ml ethanol, 95 samples total) were sent to the Australian Genome Research Facility (AGRF, Adelaide, Australia^1^) in ethanol on dry ice for DNA extraction using the DNeasy PowerLyzer PowerSoil kit (QIAGEN). DNA concentrations were quantified using a NanoDrop ND-8000 Spectrophotometer (ThermoFisher Scientific, Waltham, MA, United States). PCR amplification, amplicon purification and high-throughput sequencing of bacterial 16S V1-3 rDNA (primers 519R: GWATTACCGCGGCKGCTG, Lane et al., 1985; and 27F: AGAGTTTGATCMTGGCTCAG, Lane, 1991) were carried out at the Ramaciotti Centre for Genomics (Sydney, Australia) on an Illumina MiSeq (San Diego, CA, United States) following the Australian Marine Microbes protocol^2^.

### Bioinformatics and Statistical Analysis

DNA sequence processing and taxonomic assignment followed the Australian Marine Microbial Biodiversity Initiative workflow (Brown et al., 2018), with data presented as amplicon sequence variants, or zero-radius operational taxonomic units (zOTUs, Edgar, 2016) to maximize potential phylogenetic resolution. In brief, paired-end reads were merged, short sequences (<400 base pair, bp) and sequences containing N’s or homopolymer runs >8 bp were removed. Sequences were de-replicated and those with <4 representatives removed. Chimeras were removed and zOTUs identified using the unoise3 command (Edgar, 2016). Quality-filtered sequences were mapped to the zOTUs to create a sample-by-read abundance matrix. Taxonomy was assigned to each zOTU based on the SILVA v132 database (Yilmaz et al., 2014).

Extraction controls produced extremely faint PCR products, and preliminary analysis showed these samples had lower OTU richness than other samples (20 ± 1 and 44 ± 3 OTUs each based on an OTU table rarefied to 8500 reads), but included krill-associated bacteria, potentially due to cross-well contamination during extraction or PCR amplification. Ordination plots based on weighted UniFrac distance showed that the extraction controls were most similar to other samples with DNA extract concentrations < 0.2 ng/μl (mostly faecal and digestive gland samples), but one control clustered with higher yield samples based on unweighted UniFrac distance (Supplementary Figure S1). This reflects presence of krill-associated zOTUs in the control but in distinct proportions to actual krill tissue samples, presumably due to cross-contamination. The effect of contamination will be greatest in samples with low quantities of endogenous DNA, as exemplified in this case by the extraction controls. Hence, we conservatively excluded all samples with low DNA concentrations (<0.2 ng/μl, *n* = 13 faecal, 5 digestive gland and 1 stomach sample), although we note that removing these samples had little effect on community profiles and characteristic taxa identified for each sample type. Samples with less than 5000 reads were also removed. Lastly, OTUs present in only one sample or with five reads or less across the dataset were removed.

We examined OTU richness (alpha-diversity) for each sample type with rarefaction curves generated using the ‘iNEXT’ package (Hsieh et al., 2016) in R version 3.4.2 (R Core Team, 2017). Relationships between OTU richness (based on an OTU table rarefied to 8500 reads) and sample type were investigated using negative binomial generalized linear models (GLMs) fitted using the ‘glm.nb’ function in the ‘MASS’ R package. Pairwise-comparisons of sample types were performed using the ‘glht’ function in the ‘multcomp’ R package (Hothorn et al., 2008), with *p*-values adjusted for multiple comparisons using Tukey’s Honest Significant Difference (HSD) procedure.

Differences in bacterial community composition between sample types were explored using weighted and unweighted UniFrac distances (Lozupone and Knight, 2005) in QIIME v1.8.0 (beta_diversity_through_plots.py, Caporaso et al., 2010) based on a rarefied OTU table (8500 reads). We tested for homogeneity of multivariate dispersions between sample types using PERMDISP in QIIME (compare_categories.py). The strength of groupings was assessed using PERMANOVA allowing for differences in dispersion as per Anderson et al. (2017), with significance tested using a permutation approach. We also used PERMANOVA to test whether sex, developmental stage or trawl location (swarm) were significant factors determining community similarity for three tissue types (moults, stomach and digestive gland).

The Linear Discriminant Analysis (LDA) Effect Size (LEfSe, Segata et al., 2011) method was used to identify taxa that showed different abundances between three sets of sample classes: habitat (seawater and jar water); moults; and gastro-intestinal (digestive gland, stomach, and faeces). Default settings were used except the LDA threshold was increased to 4.0 and the α-value was reduced to 0.01 to highlight the most significant taxa discriminating between sample types.

## Results

### Alpha-Diversity

The final dataset (excluding extraction blanks and samples with low DNA yield) included 3.32 million reads from 73 samples (mean ± SD = 45464 ± 22262 reads per sample, range = 8543–138951), representing 2271 OTUs. Of these, 758 (33.4%) were exclusively associated with krill tissue or faeces, i.e., not present in either seawater or jar water samples. This is likely a conservative estimate of krill-specific OTUs as faecal and moult OTUs were likely to be present in jar water, indeed 1424 OTUs (62.7%) were exclusively associated with krill tissue, faeces or jar water.

**FIGURE 2:**
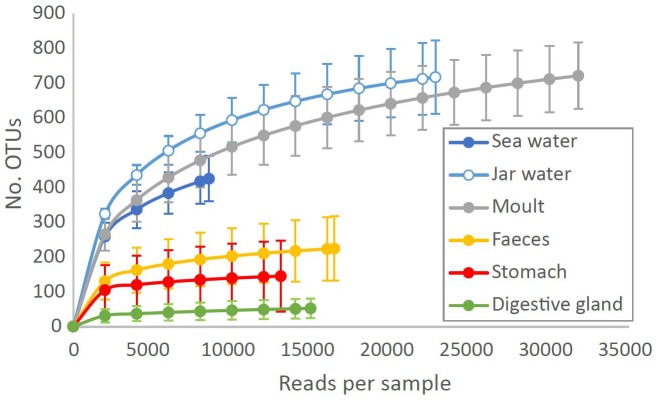
Number of bacterial OTUs observed at different sequencing depths in Southern Ocean and krill tissue samples. Data are means ± SD for each sample type at each rarefaction level, plotted to the minimum sequencing depth for each sample type.

Based on the rarefied OTU table, moult, seawater and jar water all contained a similar number of OTUs per sample (moult: 490 ± 79, seawater: 426 ± 64, jar water: 568 ± 56, *P* > 0.05). Stomach and faecal samples were less diverse (stomach: 137 ± 96, faecal: 196 ± 77), with digestive gland communities showing lower OTU richness than any other sample type (45 ± 25, *P* < 0.001). Moult communities could contain more OTUs than other tissue samples due to contamination from diverse sea (or jar) water communities. However, moult samples still had significantly higher OTU richness than stomach, digestive gland and faecal samples when all 1513 OTUs present in sea and jar water were excluded from the analysis (*P* < 0.001). Rarefaction curves show that OTU diversity is saturated for stomach, digestive gland and faecal samples with 8500 reads, whereas greater sequencing depths are required to accurately estimate diversity for moult and water samples (Figure 2).

### Differentiation Between Sample Types (Beta-Diversity)

**FIGURE 3:**
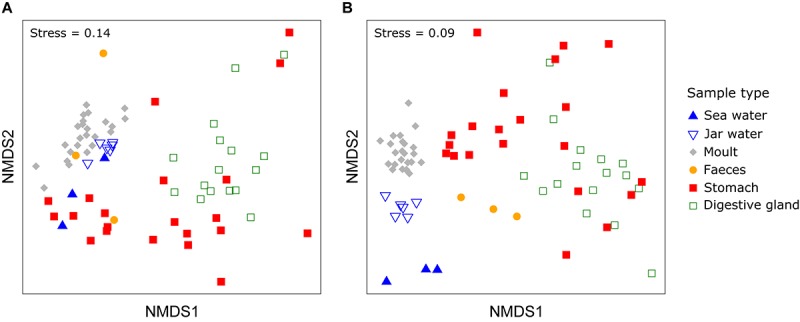
Non-metric multidimensional scaling (nMDS) plot of bacterial communities from different sample and *Euphausia superba* microhabitats using weighted **(A)** or unweighted UniFrac distance **(B)**.

PERMDISP indicated significant differences in dispersion between sample types for both weighted and unweighted UniFrac distances (weighted UniFrac: *F*_5,67_ = 10.99, unweighted UniFrac: *F*_5,67_ = 34.64, *P* < 0.001 for both). PERMANOVA allowing for differences in dispersion showed sample types supported distinct bacterial communities (weighted UniFrac: *F*_5,67_ = 10.72, *R*^2^ = 0.416, *P* < 0.001, unweighted UniFrac: *F*_5,67_ = 10.01, *R*^2^ = 0.402, *P* < 0.001, Figure 3). Pairwise comparisons of sea and jar water communities with krill moult, stomach and digestive gland communities were significant for both weighted and unweighted UniFrac distances (*P* < 0.01, Table 1), whereas faecal samples were only statistically distinct from jar water samples based on unweighted UniFrac distance (*P* = 0.01). Similarly, pairwise comparisons of the four krill sample types showed each one supported a distinct community (*P* < 0.05), with the exception of faecal versus stomach and moult communities using weighted UniFrac distance (*P* > 0.05).

**Table 1:** Results of pairwisetests using PERMANOVA allowing for differences in dispersion comparing bacterial communities between krill-associated and environmental sample types.

		Unweighted UniFrac		Weighted UniFrac	
Sample type 1	Sample type 2	*t*	*P*	*t*	*P*
Seawater	Jar water	1.83	0.011^∗^	2.13	0.024^∗^
Seawater	Moult	2.54	0.0003^∗∗^	3.15	0.0056^∗∗^
Seawater	Stomach	2.52	0.0003^∗∗^	2.78	0.0024^∗∗^
Seawater	Digestive gland	3.03	0.0018^∗∗^	3.89	0.0011^∗∗^
Seawater	Faeces	1.85	0.10	1.46	0.19
Jar water	Moult	2.72	0.0001^∗∗^	4.41	0.0001^∗∗^
Jar water	Stomach	3.50	0.0001^∗∗^	4.29	0.0001^∗∗^
Jar water	Digestive gland	4.38	0.0001^∗∗^	5.68	0.0001^∗∗^
Jar water	Faeces	2.08	0.012^∗^	1.35	0.12
Moult	Stomach	3.96	0.0001^∗∗^	4.13	0.0001^∗∗^
Moult	Digestive gland	5.20	0.0001^∗∗^	5.59	0.0001^∗∗^
Moult	Faeces	2.05	0.0003^∗∗^	1.07	0.36
Stomach	Digestive gland	1.96	0.0003^∗∗^	2.41	0.0002^∗∗^
Stomach	Faeces	1.47	0.025^∗^	1.54	0.088
Digestive gland	Faeces	1.80	0.0024^∗∗^	1.94	0.013^∗^

*Dissimilarities between communities were estimated using weighted and unweighted UniFrac distance. P-values are based on a permutation approach. ^∗^P < 0.05, ^∗∗^P < 0.01.*

**FIGURE 4:**
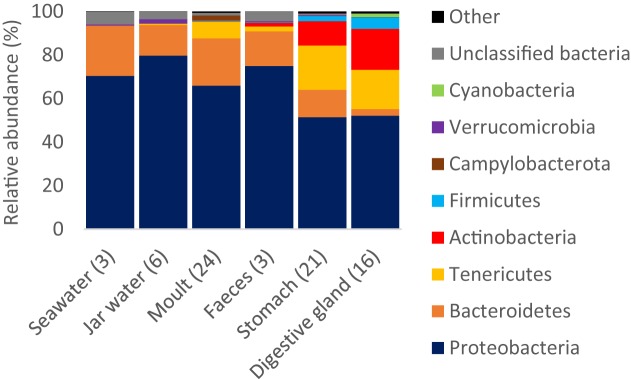
Relative read abundance of bacterial phyla by sample type. Phyla that were >1% reads in any one sample type are shown, the remaining phyla are pooled as ‘Other’. The number of replicates per sample type is shown in brackets.

Proteobacteria were >50% of reads on average across all sample types (Figure 4, see Supplementary Figure S2 for class and order level plots). Bacteroidetes were present in all samples, but showed the highest mean relative abundance in seawater (23.1%) and moult samples (21.8%). Tenericutes and Firmicutes were largely restricted to tissue samples, whereas the mean contribution of Actinobacteria was 1.7–19.0% in the three digestive tract sample types (stomach, digestive gland and faeces), but less than 1% in other samples.

Two of the three most abundant OTUs in the study were from the order Mycoplasmoidales (phylum Tenericutes, Gupta et al., 2018), and most closely related to ‘*Candidatus* Hepatoplasma,’ a genus associated with arthropod midgut (hepatopancreas) communities, known to show host-specificity and associated with survivorship of (terrestrial) isopods on low quality food (Fraune and Zimmer, 2008). Mean relative abundances were highest in digestive gland and stomach tissue (20 ± 29% and 12 ± 28%, respectively), but were highly stochastic, being absent from four stomach and two digestive gland samples, but greater than 20% of reads in 13 samples (3 stomachs, 5 digestive glands, 5 moults), and greater than 75% in two stomach and two digestive gland samples. Presence was not related to sex, developmental stage, or trawl, with all swarms including individuals where Mycoplasmoidales represented <0.5% and >10% of digestive gland reads.

### LEfSe Results

**FIGURE 5:**
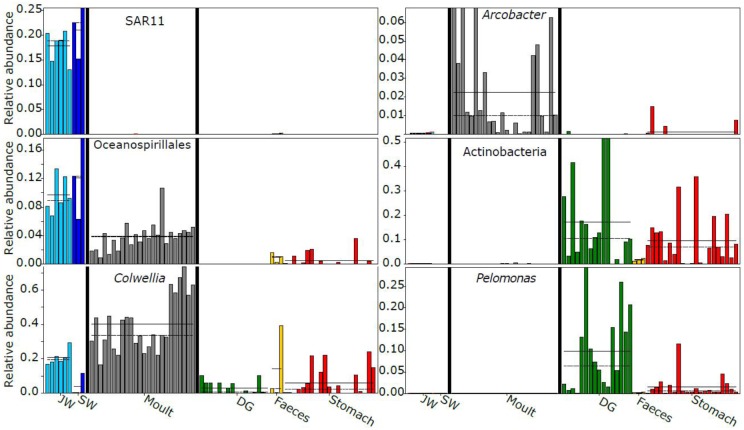
Examples of taxa displaying differential abundance between sample classes (habitat, moult or gastro-intestinal). Sample type-specific mean relative abundances are shown with a solid horizontal line, and medians with a dashed line where possible. JW, jar water; SW, seawater; DG, digestive gland.

LEfSe analysis showed 46 taxa displayed different abundances between habitat (seawater and jar water), moult and gastro-intestinal (GI, including digestive gland, stomach, and faeces) bacterial communities (*P* < 0.01, LDA > 4.0). Similar numbers of taxa were enriched in each of the three groups (habitat – 18, GI – 16, moults – 12). All five orders enriched in seawater or jar water were Proteobacteria (SAR11 and SAR86 clades, Rickettsiales, Cellvibrionales, and Oceanospirillales). SAR11 in particular had a mean relative abundance of 19 ± 4% in sea and jar water (Figure 5). Orders enriched in the krill-associated samples included members of the Campylobacterota (Campylobacterales, moults), Bacteroidetes (unclassified Bacteroidia, moults), Actinobacteria (Propionibacteriales, GI) and Firmicutes (Bacillales, GI). The mean relative abundance of *Colwellia* (Alteromonadales) was more than 40% across moult samples, but less than 10% in stomach, digestive gland and seawater samples (seawater – 4.0%, digestive gland – 3.0%, stomach – 6.0%). The genus *Pelomonas* (Betaproteobacteriales) had a mean relative abundance of 9.8 ± 9.5% in digestive gland samples, 1.5 ± 2.5% in stomach, but <0.1% in moult and water samples. The mean relative abundance of *Arcobacter* (Campylobacterales) in moult samples was ten-fold higher than any other sample type (2.3% vs. <0.2%).

### Factors Driving Krill-Associated Microbiomes

**FIGURE 6:**
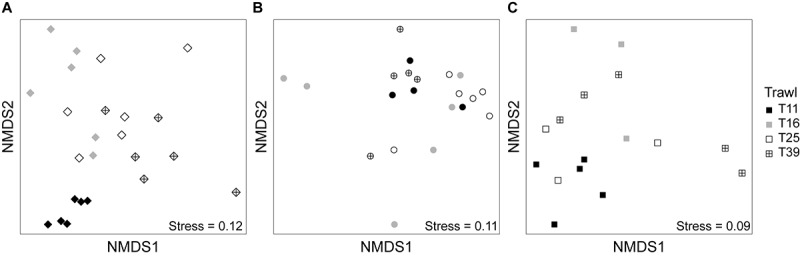
Non-metric multidimensional scaling (nMDS) plot of bacterial communities from different trawls (T11, T16, T25, and T39, see Figure 1) for moult **(A)**, stomach **(B)**, and digestive gland samples **(C)** using weighted UniFrac distance.

We performed three-way PERMANOVAs to test whether trawl location (swarm membership), developmental stage or sex were significant factors determining bacterial community similarity for three tissue types (moults, stomach and digestive gland). Trawl location explained the greatest proportion of variance and had the lowest *P*-value for each of the three tissue types (moult: *F*_3,15_ = 11.88, *R*^2^ = 0.579, *P* < 0.001, stomach: *F*_3,12_ = 1.90, *R*^2^ = 0.271, *P* = 0.06, digestive gland: *F*_3,8_ = 2.18, *R*^2^ = 0.336, *P* = 0.04, Figure 6 and Supplementary Table S1). Developmental stage was also marginally significant (*P* = 0.036) for moult communities, with a significant interaction between trawl and sex (*P* < 0.01). However, the four trawls did not have a balanced number of developmental stages or sexes (males, females, and juveniles), but tended to be dominated by females or juveniles that we could not sex, with only four males in the 24 samples. Hence, this particular experiment was not the ideal design to test whether sex or developmental stage is a key determinant of krill-associated microbiomes, especially in light of the strong effect of swarm membership.

## Discussion

In this study, we show Antarctic krill support distinct bacterial communities compared to the surrounding seawater, with each tissue representing distinct microhabitats with their own bacterial assemblages. Between 33 and 63% of the OTUs from this study represent exclusively krill-associated bacteria, suggesting krill are a major source of Southern Ocean microbial diversity. Krill also supported distinct phyla to those found in the surface seawater samples, including Actinobacteria, Campylobacterota, Firmicutes, and Tenericutes. Although Actinobacteria can represent a substantial proportion of the bacterial community in circumpolar deep water, the other three phyla do not reach relative abundances > 1% in any Southern Ocean water mass (Wilkins et al., 2013; Sow et al., unpublished data). Different tissues host distinct bacterial communities (Figures 3–5), as per the human and other microbiomes (The Human Microbiome Project Consortium et al., 2012; Llewellyn et al., 2014; Dittmer et al., 2016). Our results vastly extend the known diversity of krill-associated bacteria based on culture-dependent techniques (e.g., Donachie and Zdanowski, 1998; Gómez-Gutiérrez and Morales-Ávila, 2016), and by looking at multiple tissues from krill collected across different locations, provides the first insight into factors influencing the krill microbiota.

The Antarctic krill moult and gut microbiota show similarities to other marine microbiomes. High relative abundances of moult-associated Colwelliaceae were also found in *Calanus* copepods (Moisander et al., 2015). Some *Colwellia* species are chitinolytic (Deming et al., 1988), which may allow them to exploit the estimated 15 million tons of chitin produced in krill moults per annum (Nicol and Hosie, 1993). We found Oceanospirillales were enriched in sea and jar water compared to krill-associated communities, but were also present in all moult samples (Figure 5), as in the copepod-associated microbiome (Shoemaker and Moisander, 2015). The moult-associated *Arcobacter* OTU showed 96% identity to a Southern Ocean yeti crab episymbiont (*Kiwa* sp., Zwirglmaier et al., 2015), suggesting conservation, to some degree, of epibionts between benthic and pelagic Southern Ocean taxa. Enrichment of Burkholderiaceae in krill gastro-intestinal tissue, largely driven by *Pelomonas*, has also been observed in the copepod-associated microbiome (Shoemaker and Moisander, 2015). *Pelomonas* is also found in other marine and terrestrial arthropods (Shelomi et al., 2013; Gorokhova et al., 2015), and some *Hydra*-associated species have antifungal properties (Fraune et al., 2015).

Our results suggest some, but not all, Antarctic krill harbor a bacterial gut symbiont. Two of the most abundant OTUs in this study were from the order Mycoplasmoidales, with 83% sequence identity to *Candidatus* Hepatoplasma crinochetorum, a bacterium known to improve survival of terrestrial isopods on low quality food (Fraune and Zimmer, 2008). However, the greater similarity of the krill-associated ‘*Candidatus* Hepatoplasma’ 16S sequence to bacteria isolated from marine compared to terrestrial crustaceans (93–95% vs. 83%) suggests the marine taxa may represent a distinct clade within the order Mycoplasmoidales. If Mycoplasmoidales has a similar role in Antarctic krill, its presence could assist with survival over-winter or the transition from the euphotic to the benthic zone. The stochastic occurrence and abundance could be related to the periodic loss of the stomach during moulting, or the need to acquire the bacterium from the environment as observed for terrestrial isopods (Wang et al., 2007). Metagenomic studies could resolve whether krill gastro-intestinal tissues colonized by Mycoplasmoidales have a distinct functional profile to other krill. The most closely related sequences in GenBank have 95% identity to a bacterium from Norwegian lobster, and 93% identity to a hydrothermal vent shrimp (*Chorocaris chacei*) bacterium, raising the possibility that many marine crustaceans host species-specific Mycoplasmoidales symbionts. Future research should test whether Southern Ocean euphausiids that co-occur with Antarctic krill also host Mycoplasmoidales, and whether they are host-specific.

We found moult communities had similar richness to seawater, whereas gastro-intestinal samples had much lower diversity (Figure 2). Oriental river prawn gut microbiomes were also less diverse than corresponding lake or river water bacterial diversity (Chen et al., 2017), whereas the diversity of crayfish carapace communities were similar to and correlated with their environment (Skelton et al., 2016). Moult communities were collected within 12 h of being shed and could include bacteria that colonized after moulting, but this method has the benefit of sampling all bacteria associated with the carapace versus only surface-associated bacteria retrieved by swabbing, and that all krill are sampled at the same point in the moult cycle. Quantifying absolute microbial abundance by qPCR or flow cytometry (e.g., Vandeputte et al., 2017) would show whether low gastro-intestinal diversity is a result of low bacterial biomass, and potentially elucidate the importance of post-moult colonization.

The observed variation in moult and gastro-intestinal microbiota between trawls increases the contribution of krill-associated bacteria to total Southern Ocean microbial diversity, as each swarm supports distinct assemblages. It would be interesting in future studies to test whether diversity of moult communities in particular is driven by the ambient environment as per Skelton et al. (2016), ensuring adequate sequencing depth to reliably estimate richness of seawater communities (Figure 2). Surface samples were used to assess seawater communities, whereas the three trawls with matched surface water samples had minimum and maximum depths of 10–30 and 25–35 m, respectively. Although Southern Ocean surface waters can have distinct bacterial communities to those observed at ∼30 m depth (Signori et al., 2014), several lines of evidence suggest the surface water was representative of the krill environment and that krill support distinct bacterial communities compared to the surrounding environment. Firstly, the surface mixed layer depth near these three trawls was similar to or below the relevant maximum trawl depth (25–52 m, Bestley et al., 2018), suggesting the bacterial community should be similar at the surface and the depth of the krill swarm. Secondly, in the austral summer Antarctic krill migrate to the surface during diel vertical migration (Siegel, 2005; Taki et al., 2005), hence will be immersed in the surface water bacterial community for at least part of each day. Thirdly, the ubiquitous marine bacterial SAR11 clade represented approximately 20% of reads in all water samples, but was near-absent from all krill-associated bacterial communities (Figure 5), supporting the distinct nature of the krill microbiome.

Stomach and digestive glands potentially contained transient food-associated bacteria not permanently associated with krill, and could explain the greater dispersion among GI samples compared to other sample types (Figure 3). However, the consistent presence of taxa in GI tissue across four locations suggests permanent associations. Future research could examine gastro-intestinal bacterial communities for starved krill to confirm stable associations (e.g., Moisander et al., 2015).

## Conclusion

Antarctic krill represent a key microbial habitat in the Southern Ocean. As well as representing a phylogenetically distinct community, it is possible that specialized krill-associated assemblages contribute a distinct suite of functional traits in terms of biogeochemical cycling. Krill swarms influence Southern Ocean microbial communities not only through grazing of eukaryotic cells and release of nutrients into the water column (Arístegui et al., 2014), but also by providing distinct habitats and a means to transport microbial assemblages both horizontally and vertically. Krill swarms have been observed to migrate 215 km in 16.5 days (Kanda et al., 1982), with routine diel vertical migrations up to 200 m (Siegel, 2005; Taki et al., 2005), and quite possibly much greater. The majority of Antarctic krill biomass is found within the epipelagic zone throughout the year (0–200 m, Siegel and Watkins, 2016), but adult krill have been observed at depths up to 3500 m (Clarke and Tyler, 2008; Kawaguchi et al., 2011). Vertical migration, as well as sinking faecal pellets and moults, are likely to contribute to dispersal of krill-associated bacteria throughout the water column. Changes to Antarctic krill demographics or distribution through fishing pressure or climate-induced range shifts (Meyer et al., 2017) will also influence the composition and dispersal of Southern Ocean microbial communities. The results of this study will pave the way for future research into krill biology and ecology, bacterial symbioses, and Southern Ocean microbiology and biogeochemical cycling.

## Data Availability Statement

The datasets generated for this study can be found in the NCBI database under BioProject ID: PRJNA505226.

## Author Contributions

LC, LS, RK, and BD conceived the experiments and collected the samples. AB and LC performed the data analysis. LC wrote the manuscript. All authors contributed to discussing results and the final version of the manuscript.

## Conflict of Interest Statement

The authors declare that the research was conducted in the absence of any commercial or financial relationships that could be construed as a potential conflict of interest.

## Footnotes

1 http://www.agrf.org.au

2 http://www.bioplatforms.com/marine-microbes/

## References

[B1] Anderson M. J. Walsh D. C. I. Robert Clarke K. Gorley R. N. Guerra-Castro E. (2017). Some solutions to the multivariate Behrens-Fisher problem for dissimilarity-based analyses. *Aust. N. Z. J. Stat.* 59 57–79. 10.1111/anzs.12176

[B2] Arístegui J. Duarte C. M. Reche I. Gómez-Pinchetti J. L. (2014). Krill excretion boosts microbial activity in the Southern Ocean. *PLoS One* 9:e89391. 10.1371/journal.pone.0089391 24586744 PMC3929700

[B3] Atkinson A. Siegel V. Pakhomov E. A. Jessopp M. J. Loeb V. (2009). A re-appraisal of the total biomass and annual production of Antarctic krill. *Deep Sea Res. Part I Oceanogr. Res. Pap.* 56 727–740. 10.1016/j.dsr.2008.12.007

[B4] Bestley S. van Wijk E. Rosenberg M. Eriksen R. Corney S. Tattersall K. (2018). Ocean circulation and frontal structure near the southern Kerguelen Plateau: the physical context for the Kerguelen Axis ecosystem study. *Deep Sea Res. Part II Top. Stud. Oceanogr.* 10.1016/j.dsr2.2018.07.013

[B5] Brown M. V. van de Kamp J. Ostrowski M. Seymour J. R. Ingleton T. Messer L. F. (2018). Systematic, continental scale temporal monitoring of marine pelagic microbiota by the Australian Marine Microbial Biodiversity Initiative. *Sci. Data* 5:180130. 10.1038/sdata.2018.130 30015804 PMC6049030

[B6] Caporaso J. G. Kuczynski J. Stombaugh J. Bittinger K. Bushman F. D. Costello E. K. (2010). QIIME allows analysis of high-throughput community sequencing data. *Nat. Methods* 7 335–336. 10.1038/nmeth0510-335 20383131 PMC3156573

[B7] Chen C. Y. Chen P. C. Weng F. C. Shaw G. T. Wang D. (2017). Habitat and indigenous gut microbes contribute to the plasticity of gut microbiome in oriental river prawn during rapid environmental change. *PLoS One* 12:e0181427. 10.1371/journal.pone.0181427 28715471 PMC5513549

[B8] Clarke A. Tyler P. A. (2008). Adult Antarctic krill feeding at abyssal depth. *Curr. Biol.* 18 282–285. 10.1016/j.cub.2008.01.059 18302926

[B9] Cottrell M. T. Wood D. N. Yu L. Kirchman D. L. (2000). Selected chitinase genes in cultured and uncultured marine bacteria in the α- and γ-subclasses of the *proteobacteria*. *Appl. Environ. Microbiol.* 66 1195–1201. 10.1128/AEM.66.3.1195-1201.2000 10698791 PMC91962

[B10] Croxall J. P. Reid K. Prince P. A. (1999). Diet, provisioning and productivity responses of marine predators to differences in availability of Antarctic krill. *Mar. Ecol. Prog. Ser.* 177 115–131. 10.3354/meps177115

[B11] Deming J. W. Somers L. K. Straube W. L. Swartz D. G. MacDonell M. T. (1988). Isolation of an obligately barophilic bacterium and description of a new genus, Colwellia gen. nov. *Syst. Appl. Microbiol.* 10 152–160. 10.1016/S0723-2020(88)80030-4

[B12] Dittmer J. Lesobre J. Moumen B. Bouchon D. (2016). Host origin and tissue microhabitat shaping the microbiota of the terrestrial isopod Armadillidium vulgare. *FEMS Microbiol. Ecol.* 92:fiw063. 10.1093/femsec/fiw063 27004796

[B13] Donachie S. P. Zdanowski M. K. (1998). Potential digestive function of bacteria in krill *Euphausia superba* stomach. *Aquat. Microbial Ecol.* 14 129–136. 10.3354/ame014129

[B14] Edgar R. C. (2016). UNOISE2: improved error-correction for Illumina 16S and ITS amplicon sequencing. *bioRxiv* [Preprint]. 10.1101/081257

[B15] Fraune S. Anton-Erxleben F. Augustin R. Franzenburg S. Knop M. Schroder K. (2015). Bacteria-bacteria interactions within the microbiota of the ancestral metazoan Hydra contribute to fungal resistance. *ISME J.* 9 1543–1556. 10.1038/ismej.2014.239 25514534 PMC4478695

[B16] Fraune S. Zimmer M. (2008). Host-specificity of environmentally transmitted Mycoplasma-like isopod symbionts. *Environ. Microbiol.* 10 2497–2504. 10.1111/j.1462-2920.2008.01672.x 18833647

[B17] Gómez-Gutiérrez J. Morales-Ávila J. R. (2016). “Parasites and diseases,” in *Biology and Ecology of Antarctic krill*, ed. Siegel V. (Cham: Springer), 351–386. 10.1007/978-3-319-29279-3_10

[B18] Gorokhova E. Rivetti C. Furuhagen S. Edlund A. Ek K. Breitholtz M. (2015). Bacteria-mediated effects of antibiotics on Daphnia nutrition. *Environ. Sci. Technol.* 49 5779–5787. 10.1021/acs.est.5b00833 25850437

[B19] Gupta R. S. Sawnani S. Adeolu M. Alnajar S. Oren A. (2018). Phylogenetic framework for the phylum Tenericutes based on genome sequence data: proposal for the creation of a new order *Mycoplasmoidales* ord. nov., containing two new families *Mycoplasmoidaceae* fam. nov. and *Metamycoplasmataceae* fam. nov. harbouring *Eperythrozoon, Ureaplasma* and five novel genera. *Antonie Van Leeuwenhoek* 111 1583–1630. 10.1007/s10482-018-1047-3 29556819

[B20] Hothorn T. Bretz F. Westfall P. (2008). Simultaneous inference in general parametric models. *Biometrical J.* 50 346–363. 10.1002/bimj.200810425 18481363

[B21] Hsieh T. C. Ma K. H. Chao A. McInerny G. (2016). iNEXT: an R package for rarefaction and extrapolation of species diversity (Hill numbers). *Methods Ecol. Evol.* 7 1451–1456. 10.1111/2041-210x.12613

[B22] Kanda K. Takagi K. Seki Y. (1982). Movement of the larger swarms of Antarctic krill *Euphausia superba* population off Enderby Land during 1976-1977 season. *J. Tokyo Univ. Fish.* 68 25–42.

[B23] Kawaguchi S. Candy S. G. King R. Naganobu M. Nicol S. (2006). Modelling growth of Antarctic krill. I. Growth trends with sex, length, season, and region. *Mar. Ecol. Prog. Ser.* 306 1–15. 10.3354/meps306001

[B24] Kawaguchi S. Kilpatrick R. Roberts L. King R. A. Nicol S. (2011). Ocean-bottom krill sex. *J. Plankton Res.* 33 1134–1138. 10.1093/plankt/fbr006 21655471 PMC3109991

[B25] Lane D. J. (1991). “16S/23S rRNA sequencing,” in *Nucleic Acid Techniques in Bacterial Systematics*, eds Stachebrandt E. Goodfellow M. (Chichester, NY: John Wiley and Sons), 115–175.

[B26] Lane D. J. Pace B. Olsen G. J. Stahl D. A. Sogin M. L. Pace N. R. (1985). Rapid determination of 16S ribosomal RNA sequences for phylogenetic analyses. *Proc. Natl. Acad. Sci. U.S.A.* 82 6955–6959. 10.1073/pnas.82.20.6955 2413450 PMC391288

[B27] LeCleir G. R. Buchan A. Hollibaugh J. T. (2004). Chitinase gene sequences retrieved from diverse aquatic habitats reveal environment-specific distributions. *Appl. Environ. Microbiol.* 70 6977–6983. 10.1128/AEM.70.12.6977-6983.2004 15574890 PMC535185

[B28] Llewellyn M. S. Boutin S. Hoseinifar S. H. Derome N. (2014). Teleost microbiomes: the state of the art in their characterization, manipulation and importance in aquaculture and fisheries. *Front. Microbiol.* 5:207. 10.3389/fmicb.2014.00207 24917852 PMC4040438

[B29] Lozupone C. Knight R. (2005). UniFrac: a new phylogenetic method for comparing microbial communities. *Appl. Environ. Microbiol.* 71 8228–8235. 10.1128/AEM.71.12.8228-8235.2005 16332807 PMC1317376

[B30] Marty D. G. (1993). Methanogenic bacteria in seawater. *Limnol. Oceanogr.* 38 452–456. 10.4319/lo.1993.38.2.0452

[B31] Melvin J. E. Kawaguchi S. King R. Swadling K. M. (2018). The carapace matters: refinement of the instantaneous growth rate method for Antarctic krill *Euphausia superba* Dana, 1850 (Euphausiacea). *J. Crustacean Biol.* 38 689–696. 10.1093/jcbiol/ruy069

[B32] Meyer B. Freier U. Grimm V. Groeneveld J. Hunt B. P. V. Kerwath S. (2017). The winter pack-ice zone provides a sheltered but food-poor habitat for larval Antarctic krill. *Nat. Ecol. Evol.* 1 1853–1861. 10.1038/s41559-017-0368-3 29133903

[B33] Moisander P. H. Sexton A. D. Daley M. C. (2015). Stable associations masked by temporal variability in the marine copepod microbiome. *PLoS One* 10:e0138967. 10.1371/journal.pone.0138967 26393930 PMC4579122

[B34] Nicol S. Hosie G. W. (1993). Chitin production by krill. *Biochem. Syst. Ecol.* 21 181–184. 10.1016/0305-1978(93)90035-P

[B35] Orsi A. H. Whitworth T. Nowlin W. D. (1995). On the meridional extent and fronts of the antarctic circumpolar current. *Deep Sea Res. Part I Oceanogr. Res. Pap.* 42 641–673. 10.1016/0967-0637(95)00021-w

[B36] Proctor L. M. (1997). Nitrogen-fixing, photosynthetic, anaerobic bacteria associated with pelagic copepods. *Aquat. Microbial Ecol.* 12 105–113. 10.3354/ame012105

[B37] R Core Team (2017). *R: A Language and Environment for Statistical Computing*. Vienna: R Foundation for Statistical Computing.

[B38] Schmidt K. Atkinson A. Pond D. W. Ireland L. C. (2014). Feeding and overwintering of Antarctic krill across its major habitats: the role of sea ice cover, water depth, and phytoplankton abundance. *Limnol. Oceanogr.* 59 17–36. 10.4319/lo.2014.59.1.0017

[B39] Segata N. Izard J. Waldron L. Gevers D. Miropolsky L. Garrett W. S. (2011). Metagenomic biomarker discovery and explanation. *Genome Biol.* 12:R60. 10.1186/gb-2011-12-6-r60 21702898 PMC3218848

[B40] Shelomi M. Lo W. S. Kimsey L. S. Kuo C. H. (2013). Analysis of the gut microbiota of walking sticks (Phasmatodea). *BMC Res. Notes* 6:368. 10.1186/1756-0500-6-368 24025149 PMC3856447

[B41] Shoemaker K. M. Moisander P. H. (2015). Microbial diversity associated with copepods in the North Atlantic subtropical gyre. *FEMS Microbiol. Ecol.* 91:fiv064. 10.1093/femsec/fiv064 26077986

[B42] Siegel V. (2005). Distribution and population dynamics of *Euphausia superba*: summary of recent findings. *Polar Biol.* 29 1–22. 10.1007/s00300-005-0058-5

[B43] Siegel V. Watkins J. L. (2016). “Distribution, biomass and demography of Antarctic Krill, *Euphausia superba*,” in *Biology and Ecology of Antarctic Krill*, ed. Siegel V. (Cham: Springer), 21–100. 10.1007/978-3-319-29279-3-2

[B44] Signori C. N. Thomas F. Enrich-Prast A. Pollery R. C. Sievert S. M. (2014). Microbial diversity and community structure across environmental gradients in Bransfield Strait. Western Antarctic Peninsula. *Front. Microbiol.* 5:647. 10.3389/fmicb.2014.00647 25566198 PMC4267279

[B45] Skelton J. Geyer K. M. Lennon J. T. Creed R. P. Brown B. L. (2016). Multi-scale ecological filters shape the crayfish microbiome. *Symbiosis* 72 159–170. 10.1007/s13199-016-0469-9

[B46] Sunagawa S. Coelho L. P. Chaffron S. Kultima J. R. Labadie K. Salazar G. (2015). Structure and function of the global ocean microbiome. *Science* 348:1261359. 10.1126/science.1261359 25999513

[B47] Taki K. Hayashi T. Naganobu M. (2005). Characteristics of seasonal variation in diurnal vertical migration and aggregation of Antarctic krill (*Euphausia superba*) in the Scotia Sea, using Japanese fishery data. *CCAMLR Sci.* 12 163–172.

[B48] Tang K. W. Turk V. Grossart H. P. (2010). Linkage between crustacean zooplankton and aquatic bacteria. *Aquat. Microbial Ecol.* 61 261–277. 10.3354/ame01424

[B49] The Human Microbiome Project Consortium Huttenhower C. Gevers D. Knight R. Abubucker S. Badger J. H. (2012). Structure, function and diversity of the healthy human microbiome. *Nature* 486 207–214. 10.1038/nature11234 22699609 PMC3564958

[B50] Troussellier M. Escalas A. Bouvier T. Mouillot D. (2017). Sustaining rare marine microorganisms: macroorganisms as repositories and dispersal agents of microbial diversity. *Front. Microbiol.* 8:947. 10.3389/fmicb.2017.00947 28611749 PMC5447324

[B51] Vandeputte D. Kathagen G. D’Hoe K. Vieira-Silva S. Valles-Colomer M. Sabino J. (2017). Quantitative microbiome profiling links gut community variation to microbial load. *Nature* 551 507–511. 10.1038/nature24460 29143816

[B52] Virtue P. Kawaguchi S. McIvor J. Nicol S. Wotherspoon S. Brown M. (2010). Krill growth and condition in Western Indian Ocean sector of the Southern Ocean 30-80°E in austral summer 2006. *Deep Sea Res. Part II Top. Stud. Oceanogr.* 57 948–955. 10.1016/j.dsr2.2008.11.035

[B53] Wang Y. Brune A. Zimmer M. (2007). Bacterial symbionts in the hepatopancreas of isopods: diversity and environmental transmission. *FEMS Microbiol. Ecol.* 61 141–152. 10.1111/j.1574-6941.2007.00329.x 17506824

[B54] Wilkins D. van Sebille E. Rintoul S. R. Lauro F. M. Cavicchioli R. (2013). Advection shapes Southern Ocean microbial assemblages independent of distance and environment effects. *Nat. Commun.* 4 2457. 10.1038/ncomms3457 24036630

[B55] Yilmaz P. Parfrey L. W. Yarza P. Gerken J. Pruesse E. Quast C. (2014). The SILVA and “All-species Living Tree Project (LTP)” taxonomic frameworks. *Nucleic Acids Res* 42 D643–D648. 10.1093/nar/gkt1209 24293649 PMC3965112

[B56] Zwirglmaier K. Reid W. D. Heywood J. Sweeting C. J. Wigham B. D. Polunin N. V. (2015). Linking regional variation of epibiotic bacterial diversity and trophic ecology in a new species of Kiwaidae (Decapoda, Anomura) from East Scotia Ridge (Antarctica) hydrothermal vents. *MicrobiologyOpen* 4 136–150. 10.1002/mbo3.227 25515351 PMC4335981

