# Supplementary material for: Antarctic Krill Are Reservoirs for Distinct Southern Ocean Microbial Communities
Source: Front Microbiol. 2019 Jan 15;9:3226. doi: 10.3389/fmicb.2018.03226 (PMC6340936; doi:10.3389/fmicb.2018.03226)
Supplement: Supplementary file 1 [file Data_Sheet_1.docx]

***Supplementary material***

**Antarctic krill are reservoirs for distinct Southern Ocean microbial communities**

**Laurence J. Clarke*, Léonie Suter, Rob King, Andrew Bissett, Bruce E. Deagle**

*** Correspondence:** Laurence J. Clarke: [laurence.clarke@utas.edu.au](mailto:laurence.clarke@utas.edu.au)

**Figure S1.** Non-metric multidimensional scaling (nMDS) plot of bacterial communities from DNA extraction blanks, environmental and Antarctic krill (*Euphausia superba*) microhabitats using weighted (A) or unweighted UniFrac distance (B). Samples with DNA extraction yields less than 0.2 ng/μL are shown as open symbols. Extraction blank controls are most similar to digestive gland samples with low DNA extraction yields with weighted UniFrac distance, but more similar to higher yield samples with unweighted UniFrac distance, presumably due to cross-contamination. Faecal samples with low DNA yields are widely dispersed over both ordinations, suggesting a low signal-to-noise ratio. All samples with low DNA concentrations (<0.2 ng/μl) were excluded from the final analysis.

**A B**

**Figure S2.** Relative read abundance of bacterial classes (A) and orders (B) by sample type. Classes that were >1% reads or orders that were >2% reads in any one sample type are shown, the remaining taxa are pooled as ‘Other’.

**Table S1.** Summary of PERMANOVA results testing the strength and significance of trawl location (swarm membership), developmental stage and sex on Antarctic krill bacterial communities from the moult, stomach and digestive gland, based on weighted UniFrac distance. Trawl location explained the greatest proportion of variance and had the lowest *P*-value for each of the three tissue types.

|  | D.f. | Sums of squares | Mean squares | *F* | *R*^2^ | Pr(>*F*) |
| --- | --- | --- | --- | --- | --- | --- |
| **Moult**  Trawl | 3 | 1.981 | 0.660 | 11.885 | 0.579 | 0.001 |
| Developmental stage | 2 | 0.251 | 0.126 | 2.262 | 0.074 | 0.036 |
| Sex | 1 | 0.087 | 0.087 | 1.567 | 0.025 | 0.18 |
| Trawl:Developmental stage | 1 | 0.050 | 0.050 | 0.894 | 0.015 | 0.49 |
| Trawl:Sex | 1 | 0.217 | 0.217 | 3.899 | 0.063 | 0.007 |
| Residuals | 15 | 0.833 | 0.056 |  | 0.244 |  |
| Total | 23 | 3.419 |  |  | 1.000 |  |
|  |  |  |  |  |  |  |
| **Stomach** |  |  |  |  |  |  |
| Trawl | 3 | 3.224 | 1.075 | 1.898 | 0.271 | 0.06 |
| Developmental stage | 2 | 0.671 | 0.336 | 0.593 | 0.056 | 0.82 |
| Sex | 1 | 0.101 | 0.101 | 0.179 | 0.009 | 0.99 |
| Trawl:Developmental stage | 1 | 1.038 | 1.038 | 1.833 | 0.087 | 0.12 |
| Trawl:Sex | 1 | 0.087 | 0.087 | 0.154 | 0.007 | 0.99 |
| Residuals | 12 | 6.793 | 0.566 |  | 0.570 |  |
| Total | 20 | 11.915 |  |  | 1.000 |  |
|  |  |  |  |  |  |  |
| **Digestive gland** |  |  |  |  |  |  |
| Trawl | 3 | 1.912 | 0.637 | 2.177 | 0.336 | 0.04 |
| Developmental stage | 2 | 0.941 | 0.471 | 1.607 | 0.165 | 0.16 |
| Sex | 1 | 0.233 | 0.233 | 0.796 | 0.041 | 0.54 |
| Trawl:Sex | 1 | 0.260 | 0.260 | 0.888 | 0.046 | 0.47 |
| Residuals | 8 | 2.343 | 0.293 |  | 0.412 |  |
| Total | 15 | 5.690 |  |  | 1.000 |  |
